# Serial passage of PDCoV in cell culture reduces its pathogenicity and its damage of gut microbiota homeostasis in piglets

**DOI:** 10.1128/msystems.01346-23

**Published:** 2024-02-13

**Authors:** Yunfei Zhang, Lulu Si, Junlong Gao, Xiangli Shu, Congrui Qiu, Yue Zhang, Shaopo Zu, Hui Hu

**Affiliations:** 1 The College of Veterinary Medicine, Henan Agricultural University, Zhengzhou, Henan, China; 2 Key Laboratory for Animal-derived Food Safety of Henan Province, Zhengzhou, Henan, China; 3 Ministry of Education Key Laboratory for Animal Pathogens and Biosafety, Zhengzhou, Henan, China; The University of Hong Kong, Pok Fu Lam, Hong Kong

**Keywords:** porcine deltacoronavirus (PDCoV), serial passage, genetic variation, pathogenicity, gut microbiota

## Abstract

Porcine deltacoronavirus (PDCoV) is an enteropathogenic coronavirus that mainly causes diarrhea in suckling piglets, and also has the potential for cross-species transmission. However, there are still no commercial vaccines available to prevent and control PDCoV infection. In this study, PDCoV strain HNZK-02 was serially propagated *in vitro* for up to 150 passages and the amino acid changes have mainly occurred in the S protein during serial passage which caused structure change. PDCoV HNZK-02-passage 5 (P5)-infected piglets exhibited acute and severe watery diarrhea, an obvious intestinal damage, while the piglets infected with PDCoV HNZK-02-P150 showed no obvious clinical signs, weak intestinal lesions, and lower viral loads in rectal swabs and various tissues. Compared with the PDCoV HNZK-02-P5 infection, HNZK-02-P150 infection resulted in a decrease in intestinal mucosal permeability and pro-inflammatory cytokines. Moreover, PDCoV HNZK-02-P5 infection had significantly reduced bacterial diversity and increased relative abundance of opportunistic pathogens, while PDCoV HNZK-02-P150 infection did not significantly affect the bacterial diversity, and the relative abundance of probiotics increased. Furthermore, the alterations of gut microbiota were closely related to the change of pro-inflammatory factor. Metagenomics prediction analysis demonstrated that HNZK-02-P150 modulated the tyrosine metabolism, Nucleotide-binding and oligomerization domain (NOD)-like receptor signaling pathway, and lipopolysaccharide biosynthesis, which coincided with lower inflammatory response and intestinal permeability in the piglets infected with HNZK-02-P150. In conclusion, the PDCoV HNZK-02 was successfully attenuated by serial passage *in vitro*, and the changes of S gene, metabolic function, and gut microbiota may contribute to the attenuation. The PDCoV HNZK-02-P150 may have the potential for developing live-attenuated vaccine.

**IMPORTANCE:**

Porcine deltacoronavirus (PDCoV) is an enteropathogen causing severe diarrhea, dehydration, and death in nursing piglets, devastating great economic losses for the global swine industry, and has cross-species transmission and zoonotic potential. There are currently no approved treatments or vaccines available for PDCoV. In addition, gut microbiota has an important relationship with the development of many diseases. Here, the PDCoV virulent HNZK-02 strain was successfully attenuated by serial passage on cell cultures, and the pathogenesis and effects on the gut microbiota composition and metabolic function of the PDCoV HNZK-02-P5 and P150 strains were investigated in piglets. We also found the genetic changes in the S protein during passage *in vitro* and the gut microbiota may contribute to the pathogenesis of PDCoV, while their interaction molecular mechanism would need to be explored further.

## INTRODUCTION

Coronaviruses (CoVs) are enveloped, single-stranded positive-sense RNA viruses and are categorized into four genera, *Alphacoronavirus* (α-CoV), *Betacoronavirus* (β-CoV), *Gammacoronavirus*, and *Deltacoronavirus* (δ-CoV) (1); they cause mild to severe respiratory and gastrointestinal diseases in avian and mammal species (1). Severe acute respiratory syndrome coronavirus (SARS-CoV), SARS-CoV-2, and Middle East respiratory syndrome CoV, which belong to the β-CoVs causing large-scale pandemics in humans in 2003, 2012, and 2019, respectively, resulting in significant morbidity and mortality in the human population (2, 3). In addition to infecting humans, there were six different species of CoVs that cause diseases in pigs: the α-CoVs transmissible gastroenteritis virus (TGEV), porcine respiratory coronavirus, swine enteric alphacoronavirus, porcine epidemic diarrhea virus (PEDV), β-CoV porcine hemagglutinating encephalomyelitis virus, and δ-CoV porcine deltacoronavirus (PDCoV) (4). As one of the most economically important pathogens, PEDV was first described in England in 1971 (5). In 2010, highly pathogenic strain of PEDV, belonging to the GII genotype, emerged in China and spread rapidly across the whole country, with 100% mortality rate in suckling piglets (6, 7). In 2013, the highly PEDV variant also emerged in the USA (8). After that, the highly pathogenic PEDV with the GII genotype became the dominant strain worldwide and currently circulates in pig farms, which causes huge losses to the pig industry worldwide.

PDCoV was first detected in swine feces in Hong Kong, China, in 2012 (9) and the outbreak of PDCoV in swine herds was reported in 2014 in the USA (10, 11). The clinical symptom of PDCoV was similar to the diseases caused by PEDV and TGEV, mainly causing severe diarrhea, vomiting, and death in piglets. The co-infections of PDCoV with PEDV and/or TGEV were highly prevalent in pig herds (4, 12, 13). To date, PDCoV has the capacity to infect cells from a variety of species including porcine, human, mice, chicken, calf, and feline *in vitro* (14–16). In the artificial inoculation studies, PDCoV has been proven to have a wide host range. The calves, chickens, mice, and turkeys showed certain susceptibility to PDCoV, but no obvious clinical symptoms after inoculation (17–20). Notably, PDCoV was first detected in human and isolated from the Haitian children of acute febrile illness, suggesting that there exists the risk of transmission among the human population (21). However, there are no effective vaccines or antiviral agents to control PDCoV infection. Therefore, tracking the genetic dynamics in PDCoV is necessary to develop a safe and effective PDCoV vaccine.

So far, the vaccines are the most effective medical interventions among the various methods used to impede viral transmission (22). The virus which retains the replication-competent *in vitro* and *in vivo*, but does not cause disease, is used to make the live-attenuated vaccines. The live-attenuated vaccine could induce the cellular and humoral immune responses (22, 23). Many attenuated-CoVs strains have been obtained using the cell passage, such as PEDV (24), infectious bronchitis virus (IBV) (25), and TGEV (26), which have laid a foundation for the live-attenuated vaccines. Several research groups have obtained the attenuated PEDV strain applying this traditional method in Vero cells, and the live-attenuated vaccine was widely used to prevent and control PEDV and TGEV in China (27). While the mutations of nucleotides and/or amino acid often occur during serial passaging of CoVs on cell cultures, and these mutations may correlate with viral attenuation. Previous studies showed that the deletion of 197 amino acids in the N-terminal of S gene could attenuate PEDV (28). The attenuated GD (aGD) strain could effectively protect chickens against IBV, and replacing the S gene of aGD strain alone could reduce significant pathogenicity of GD strain by a reverse genetic system (25). These results indicated that the mutation of S gene played an important role in the process of attenuation of CoVs. But the genetic variation of whole genome and the corresponding pathogenicity of PDCoV during serial passaging in cell culture were largely unknown.

Gut microbiota and its metabolites play an important role in maintaining intestinal homeostasis, and are related to the occurrence and development of many diseases (29). The commensal microbiota can compete with receptors and enteric nutrients, and produce antimicrobial compounds (30–32). In turn, when the homeostasis of gut microbiota is affected by pathogens, such as rotavirus, astrovirus, and SARS-CoV-2, the number of conditional pathogenic bacteria can increase while the number of beneficial bacteria can decrease, resulting in inflammation or diarrhea (33–35). The disturbance in gut microbiota increases the permeability of the intestinal barrier and reduces the immunity of the intestinal mucosa (36). Interestingly, several studies have reported that the gut bacteria can affect the vaccine efficacy. Gut microbiota could stimulate the Toll-like receptor (TRL)5 to enhance the immunogenicity of vaccines (37), and the metabolites produced by the gut microbiota influences vaccine response to intranasal vaccination with cholera toxin (38). Our previous study showed that PDCoV infection could alter the composition of microbiota and reduce the diversity of bacteria in the colon and feces of infected piglets (39); however, little is known regarding the composition of gut microbiota in the colon of the piglet infected with virulent and attenuated strains of PDCoV.

In our study, the PDCoV HNZK-02 was successfully attenuated by serial passage in LLC-PK cell and the post-passage biological and genetic characteristics were analyzed. The pathogenicities and effects on the gut microbiota composition of the PDCoV virulent strain HNZK-02-passage 5 (P5) and the attenuated strain P150 were investigated in piglets. Our data highlighted that the attenuation of pathogenicity (high-passage variant) and the mutual regulation of viral infection and gut microbiota, which laid a foundation for the develop potential candidate vaccine strains to prevent PDCoV.

## RESULTS

### Biological characteristics of PDCoV HNZK-02 variants during serial passage

**Fig 1:**
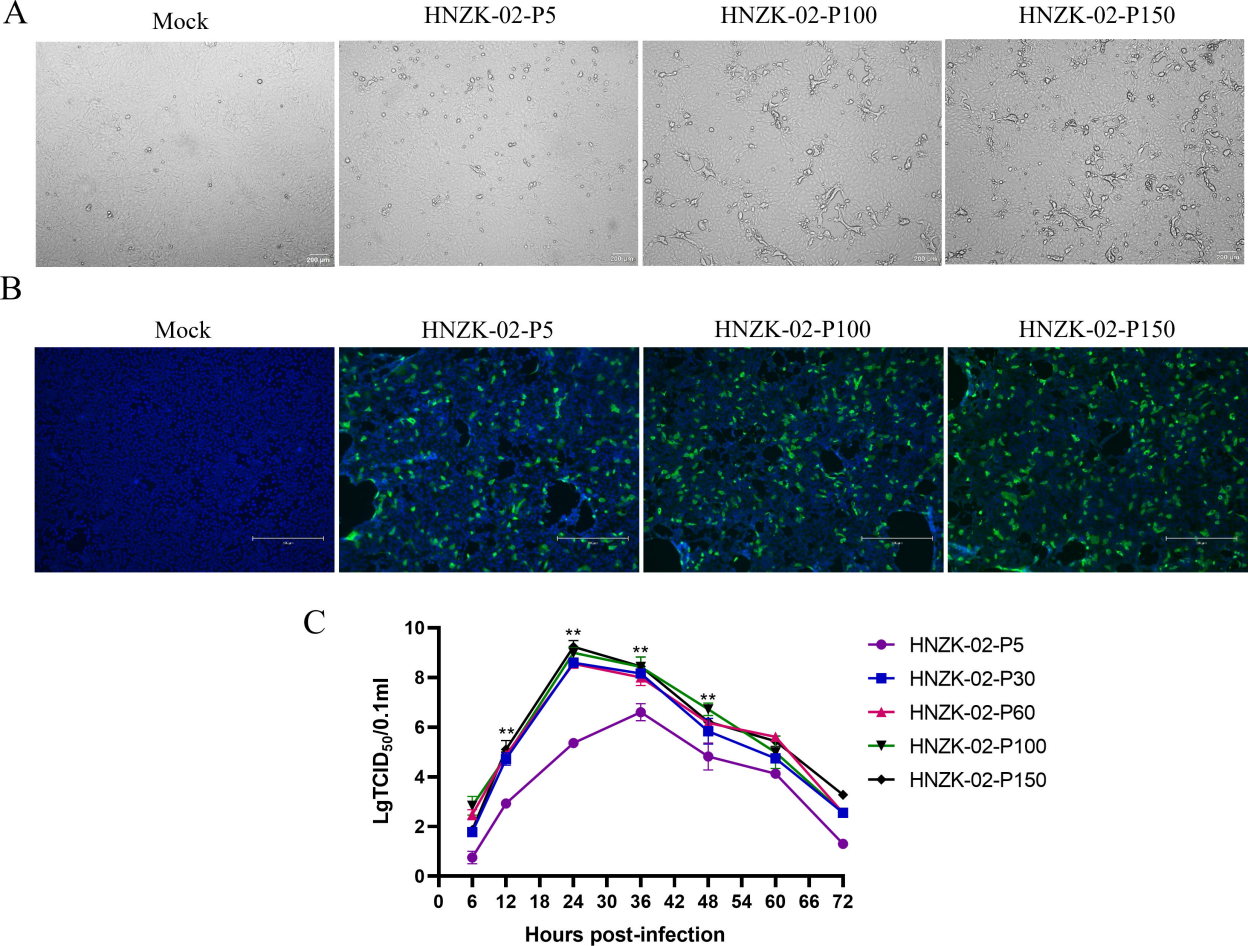
Biological characterization of PDCoV HNZK-02 strains during serial passage in LLC-PK cells. (**A**) The cytopathic effect of LLC-PK cells infected with PDCoV HNZK-02 variants P5, P100, and P150 (MOI = 0.01). (**B**) Detection of PDCoV HNZK-02 variants P5, P100, and P150 in LLC-PK by IF staining using anti-PDCoV-specific monoclonal antibody. (**C**) LLC-PK cell monolayers were infected with PDCoV HNZK-02 variants P5, P100, and P150 at an MOI of 0.01. Cell cultures were harvested at 6, 12, 24, 36, 48, 60, and 72 hpi and titrated with 50% tissue culture infectious dose assays.

To analyze the changes of biological characteristics in PDCoV HNZK-02 during the serial passage *in vitro*, the LLC-PK cells were inoculated with PDCoV HNZK-02 variants P5, P30, P60, P100, and P150 at a multiplicity of infection (MOI) of 0.01. The morphological changes were characterized by enlarged and rounded cells that inoculated with the PDCoV HNZK-02-P30, P60, P100, and P150 within 18 h. By contrast, the LLC-PK cells developed visible cytopathic effect (CPE) inoculated with PDCoV HNZK-02-P5 at 24 h post-inoculation (hpi), 6 h later than those inoculated with PDCoV HNZK-02-P30, P60, P100, and P150 (Fig. 1A). The virus was confirmed using the anti-PDCoV N-specific monoclonal antibody (prepared in our lab) (40). The infected cells showed large numbers of Immunofuorescence (IF)-stained cells, and in the negative control cells, there was no green fluorescence observed (Fig. 1B).

To determinate the growth characteristics of the PDCoV during serial passage, the PDCoV HNZK-02-P5, P30, P60, P100, and P150 strains (MOI = 0.01) were inoculated on the LLC-PK cells. We harvested the mixture of the cells and supernatants after infected 6, 12, 24, 36, 48, 60, and 72 h, respectively. The virus titer was tested by 50% tissue culture infectious dose (TCID_50_) assays. The virus titer of the low-passage variant (P5) peaked (6.8 lgTCID_50_/0.1 mL) at 36 hpi, whereas the virus titers of the high-passage variants (P30, P60, P100, and P150) reached the maximum titers of 8.5 lgTCID_50_/0.1 mL, 8.67 lgTCID_50_/0.1 mL, 9.0 lgTCID_50_/0.1 mL, and 9.2 lgTCID_50_/0.1 mL at 24 hpi, respectively (Fig. 1C). In conclusion, these results confirmed that the sensitivity and adaptability of the PDCoV HNZK-02 strain gradually increased during serial passages in LLC-PK cells.

### Phylogenetic analysis of complete genomes of PDCoV HNZK-02

**Fig 2:**
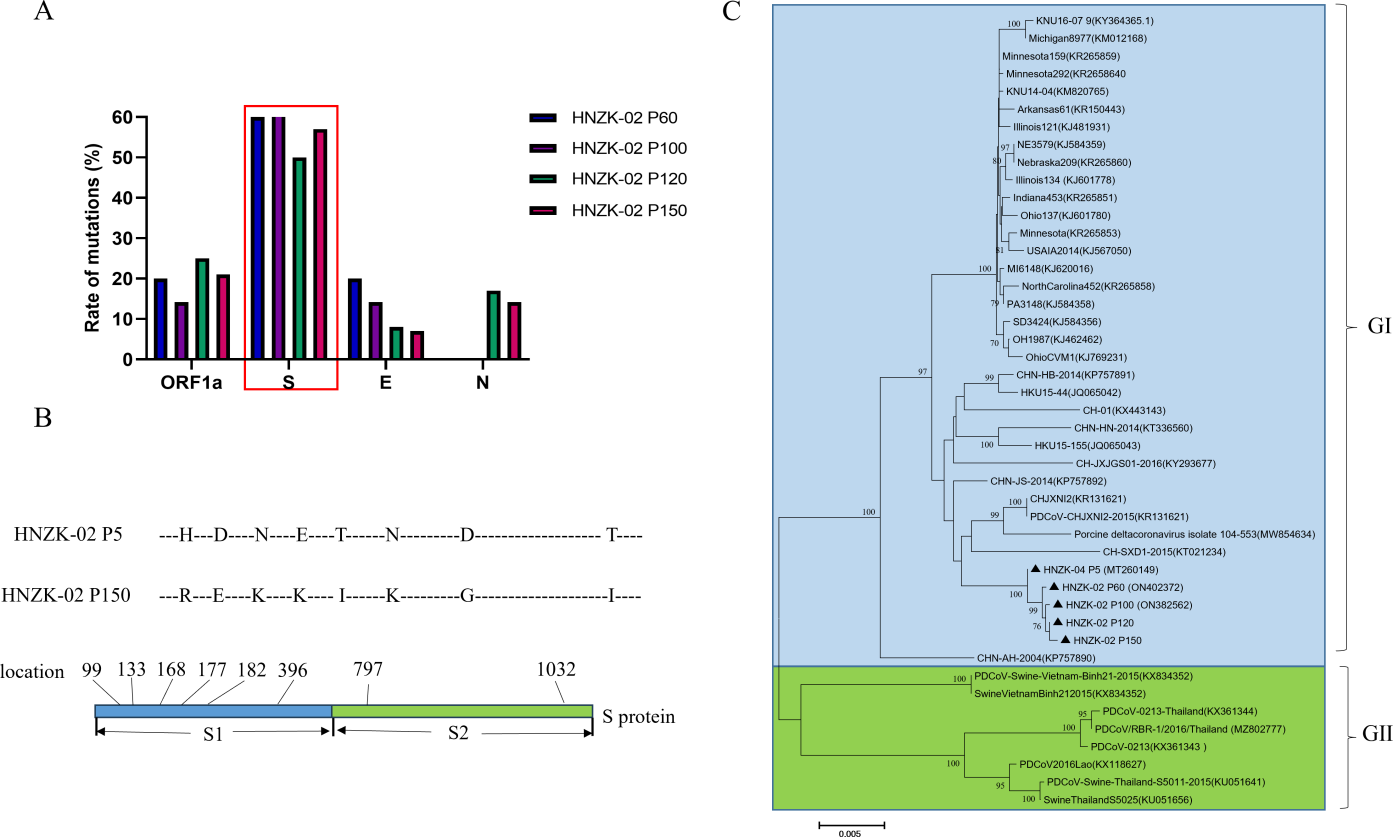
Phylogenetic analysis of PDCoV HNZK-02 cell-passaged strains and sequence alignments of the S genes of PDCoV HNZK-02 at selected passages. (**A**) The rate of amino acid mutation in the corresponding protein, including ORF1a, S, E, and N. (**B**) Sequence alignments of the amino acids of PDCoV HNZK-02-P5 and P150 S proteins were performed using MegAlign software. (**C**) The phylogenetic tree was constructed from the aligned nt sequences using the neighbor-joining method with MEGA 6.06 software (http://www.megasoftware.net) based on the whole S genes from PDCoV HNZK-02 strains P5, P60, P100, P120, and P150 with other PDCoV strains obtained from GenBank. Bootstrap values were calculated with 1,000 replicates. The black triangles indicate the PDCoV strains that were identified by our laboratory.

To further analyze the genetic variation in the PDCoV HNZK-02 during serial passage, the complete genomes of cell culture adapted were sequenced and analyzed. These results showed that compared to the genomic sequence of PDCoV HNZK-02-P5 (41), the other passaged variants (P60, P100, P120, and P150) had 5, 7, 12, and 14 amino acid (Aa) changes, respectively. In addition, in the different PDCoV proteins (ORF1a, S, E, M, NS7, and N), there were different degrees of Aa changes. There were no Aa changes in the M and NS6 proteins. The PDCoV HNZK-02-P120 and P150 showed the same numbers of Aa changes in ORF1a and E proteins, which were 3 and 1 Aa changes, respectively. The number of changes in S protein increased during their serial propagation, reaching the peak change (8 Aa) in P150. The Aa changes in PDCoV HNZK-02 variants were mainly concentrated in the S protein, with high change rates ranging from 50% to 71.4% of the total changed Aa (Fig. 2A). Of note, in the 8 Aa mutations of the S protein, there are six mutations that occurred in the S1 protein, and two occurred in the S2 protein (Fig. 2B), including His changed to Arg at position 99, Asp changed to Glu at position 133, Asn changed to Lys at position 168, Glu changed to Lys at position 177, Thr changed to Ile at position 182, Asn changed to Lys at position 396, Asp changed to Gly at position 797, and Thr changed to Ile at position 1032.

Simultaneously, the phylogenetic analyses were performed using the S gene of the PDCoV HNZK-02 strain and other 40 PDCoV strains obtained from the National Center for Biotechnology Information (NCBI). These PDCoV strains were classified into two distinct clusters (GI and GII). The GI cluster included the China, South Korea, Zambia, and USA PDCoV strains and the cell culture-adapted HNZK-02-P5, P60, P100, P120, and P150. The GII cluster included Thailand and Vietnam strains (Fig. 2C).

### Structural analysis of S proteins of PDCoV HNZK-02 variants

**Fig 3:**
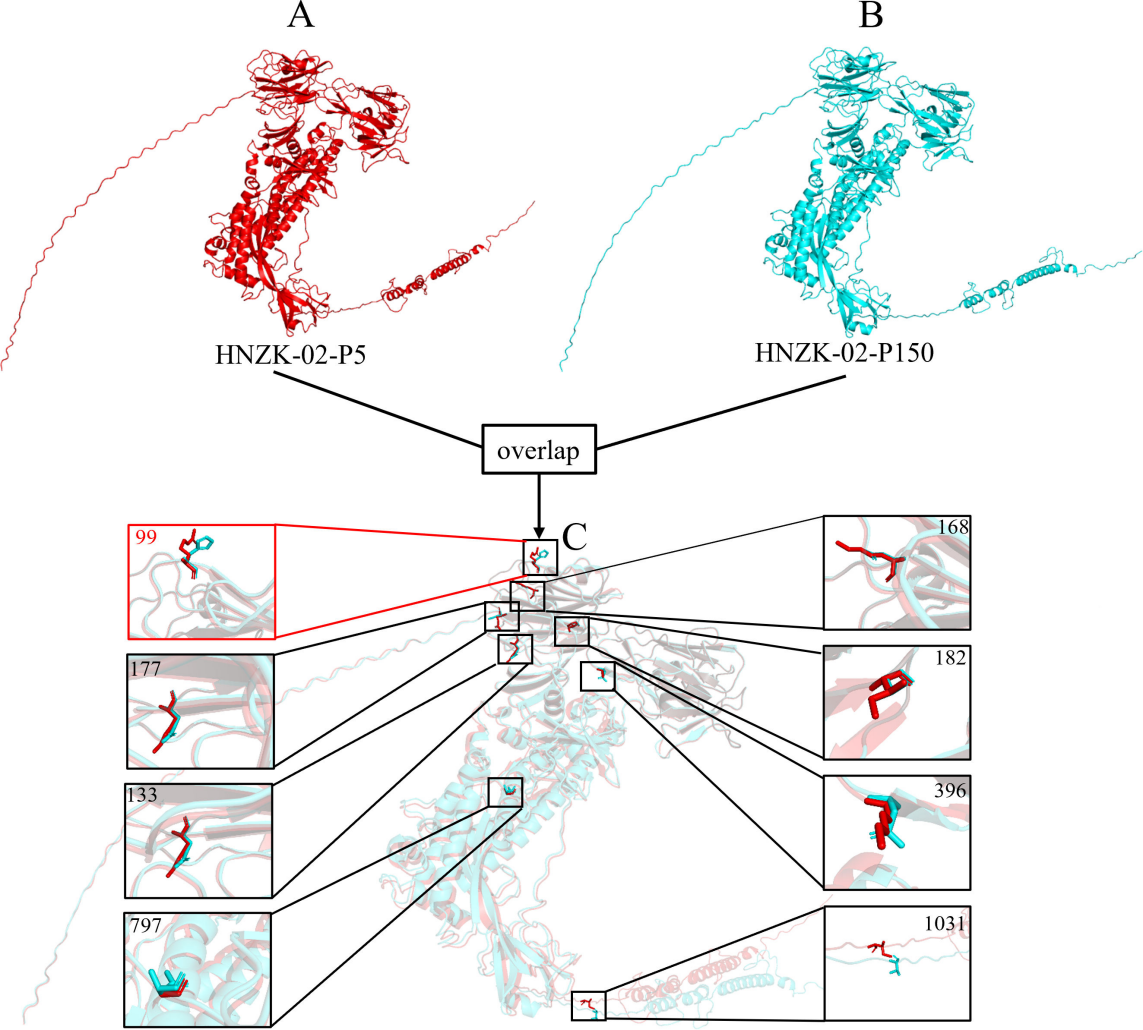
Structural analysis of the S proteins of PDCoV HNZK-02-P5 and P150 strains. The different colors were used to represent the predicted structures of S protein. Red stands for HNZK-02-P5; blue stands for HNZK-02-P150. (**A**) The predicted overall structure of S protein of HNZK-02-P5. (**B**) The predicted overall structure of S protein of HNZK-02-P150. (**C**) Structural overlap of the S proteins of PDCoV HNZK-02-P5 and P150.

Considering the obvious Aa mutations in S proteins of cell-passaged PDCoV HNZK-02 strains, the three-dimensional structures of S proteins of PDCoV HNZK-02-P5 and P150 were predicted and analyzed using PHYRE2 and PyMol software. The predicted three-dimensional structures of the S proteins of PDCoV HNZK-02-P150 have similar overall structures when compared with PDCoV HNZK-02-P5 (Fig. 3A and B). However, the monomer structural overlap of the two S proteins showed that the partial mutation (location 99) in the S1 of PDCoV HNZK-02-P150 caused its structure to change from extended strand to random coil (Fig. 3C).

### The clinical signs in 8-day-old piglets infected with PDCoV HNZK-02 variants

**Fig 4:**
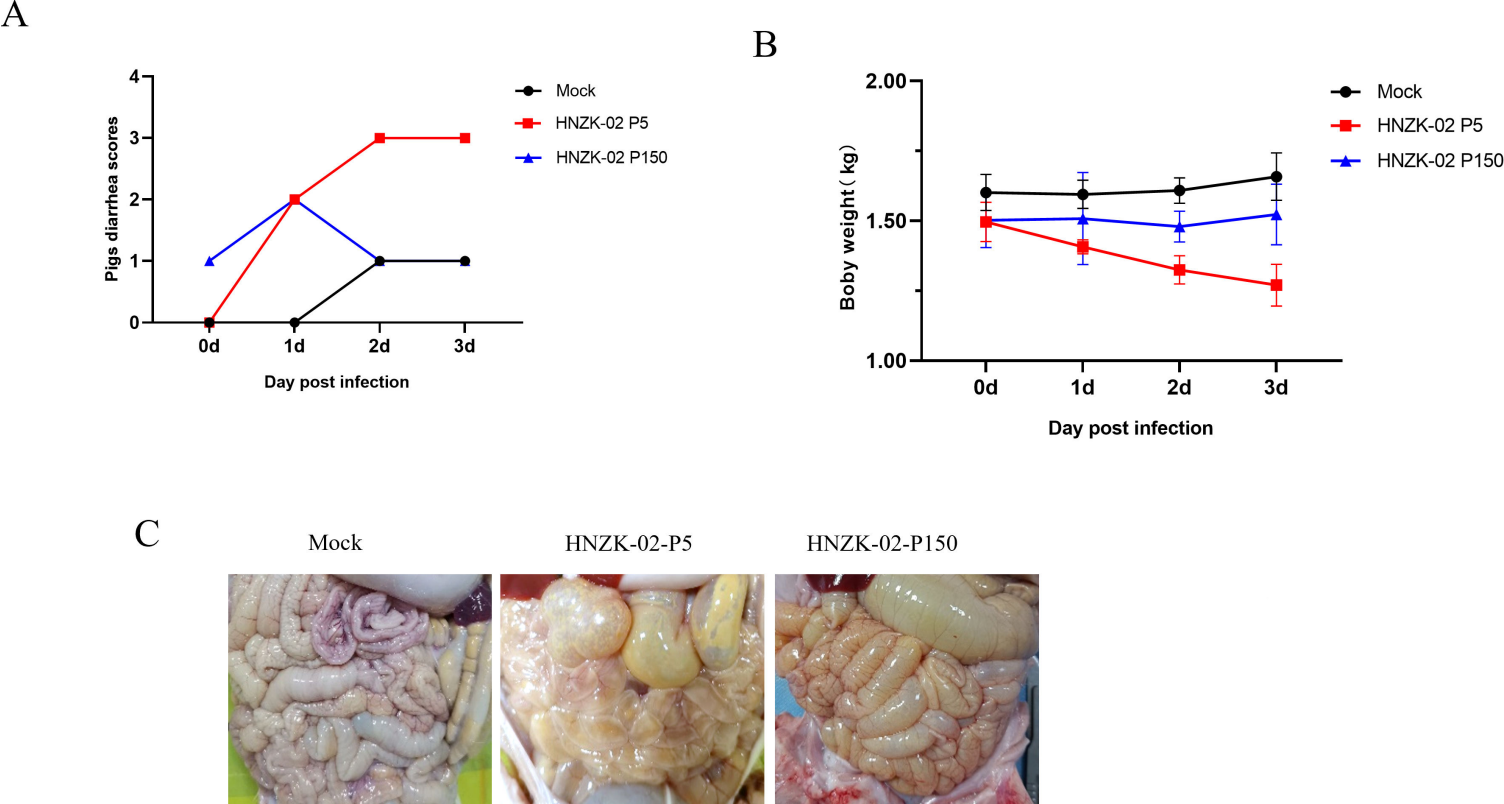
Pathogenicity analysis of piglets infected with HNZK-02-P5 or HNZK-02-P150. (**A**) Diarrhea was scored as follows: 0 = normal feces, 1 = soft but formed feces, 2 = semi-fluid feces, and 3 = watery diarrhea, with scores of 2 or more considered diarrhea. (**B**) The average body weight changes in each group. (**C**) Intestinal changes of piglets infected with PDCoV.

To analyze the change in virulence of the PDCoV HNZK-02 strain changed during its serial passage in LLC-PK cells, the 8-day-old conventional piglets were challenged with PDCoV P5 and P150 at a dose of 1 × 10^8^ TCID_50_/head via oral. These results showed that the piglets from the control groups had no obvious clinical symptoms. Acute onset of yellow watery diarrhea was observed in the group infected with PDCoV HNZK-02-P5 at 20 hpi. In the group infected with PDCoV HNZK-02-P150, one piglet had transient semiliquid feces at 2 days post-inoculation (dpi), and the other piglets showed no obvious signs (Fig. 4).

In addition, the daily body weight of each piglet was monitored, and the weights of all the piglets in the control group gradually increased throughout the experiment, as expected. The body weight of the piglets infected with PDCoV HNZK-02-P5 was significantly reduced when compared with the control piglets. By contrast, the body weights of piglets infected with PDCoV HNZK-02-P150 remained relatively stable (Fig. 4B). Altogether, the piglets infected with PDCoV HNZK-02-P150 had no obvious clinical symptoms.

### Viral RNA detection in the fecal samples and tissues of PDCoV HNZK-02-P5- and P150-infected piglets

**Fig 5:**
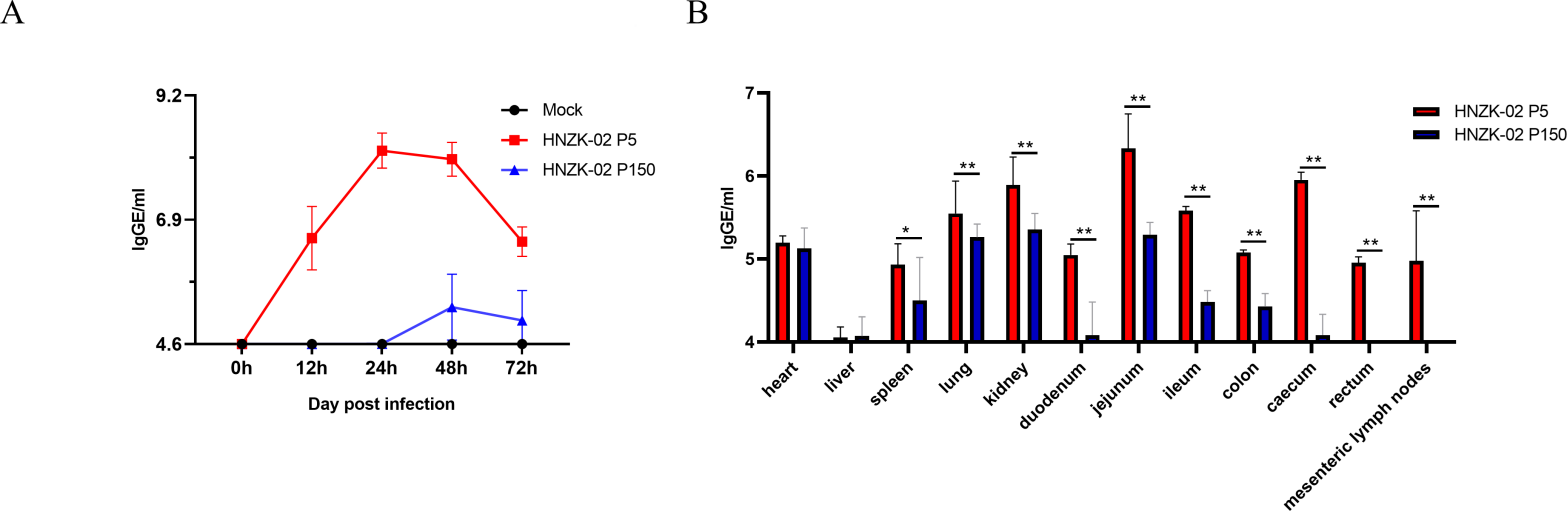
Fecal viral shedding and viral distribution in various tissues of the PDCoV HNZK-02-P5- or HNZK-02-P150-infected-pigs. Viral shedding in feces (**A**) and viral distribution in various tissue (**B**) were detected in some organs using qRT-PCR. The asterisk (*) indicates a significant difference between PDCoV HNZK-02-P5 or HNZK-02-P150 (*P < 0.05; **P < 0.01).

The fecal viral shedding in PDCoV-inoculated piglets was determined by using quantitative real-time reverse-transcription PCR (qRT-PCR) (42) at 12, 24, 48, and 72 hpi. Consistent with the clinical signs, the PDCoV viral RNA level was significantly lower in P150-inoculated piglets compared to the P5-inoculated piglets. In the group infected with PDCoV HNZK-02-P5, the PDCoV viral RNA could be detected at 12 hpi, peaked on 24 hpi (8.2 lgGE/mL), and then decreased gradually thereafter on 48 hpi. In the group infected with PDCoV HNZK-02-P150, the viral RNA could be detected at 24 hpi (5.3 lgGE/mL), and then decreased gradually thereafter (Fig. 5A).

To explore the PDCoV distribution in the organs of the piglets infected with PDCoV HNZK-02-P5 and P150, tissues from the mesenteric lymph nodes, different viscera (heart, liver, lung, spleen, and kidney), and different segments of the intestines (including the ileum, jejunum, cecum, and colon) were collected and tested. As shown in Fig. 5B, all the segments of the intestine, lung, spleen, and kidney showed high viral loads in the PDCoV HNZK-02-P5-inoculated piglets, which were significantly higher than those in the the PDCoV HNZK-02-P150-inoculated piglets. Taken together, these results indicated that the pathogenicity of PDCoV HNZK-02 to 8-day-old newborn piglets gradually decreased after serial passage *in vitro*.

### Histopathological observations

All the piglets were euthanized at 3 dpi, and the pathogenicities of the PDCoV HNZK-02-P5 and HNZK-02-P150 were evaluated systematically in suckling piglets by the pathological and histological examinations. The PDCoV HNZK-02-P5-inoculated piglets showed obvious intestinal lesions characterized by transparent, thin-walled, gas-distended dilatation and accumulation of yellow fluids (Fig. 4C). While the piglets infected with PDCoV HNZK-02 P150 showed slight flatulence in the intestinal lumen. No significant lesions were found in uninfected piglets.

**Fig 6:**
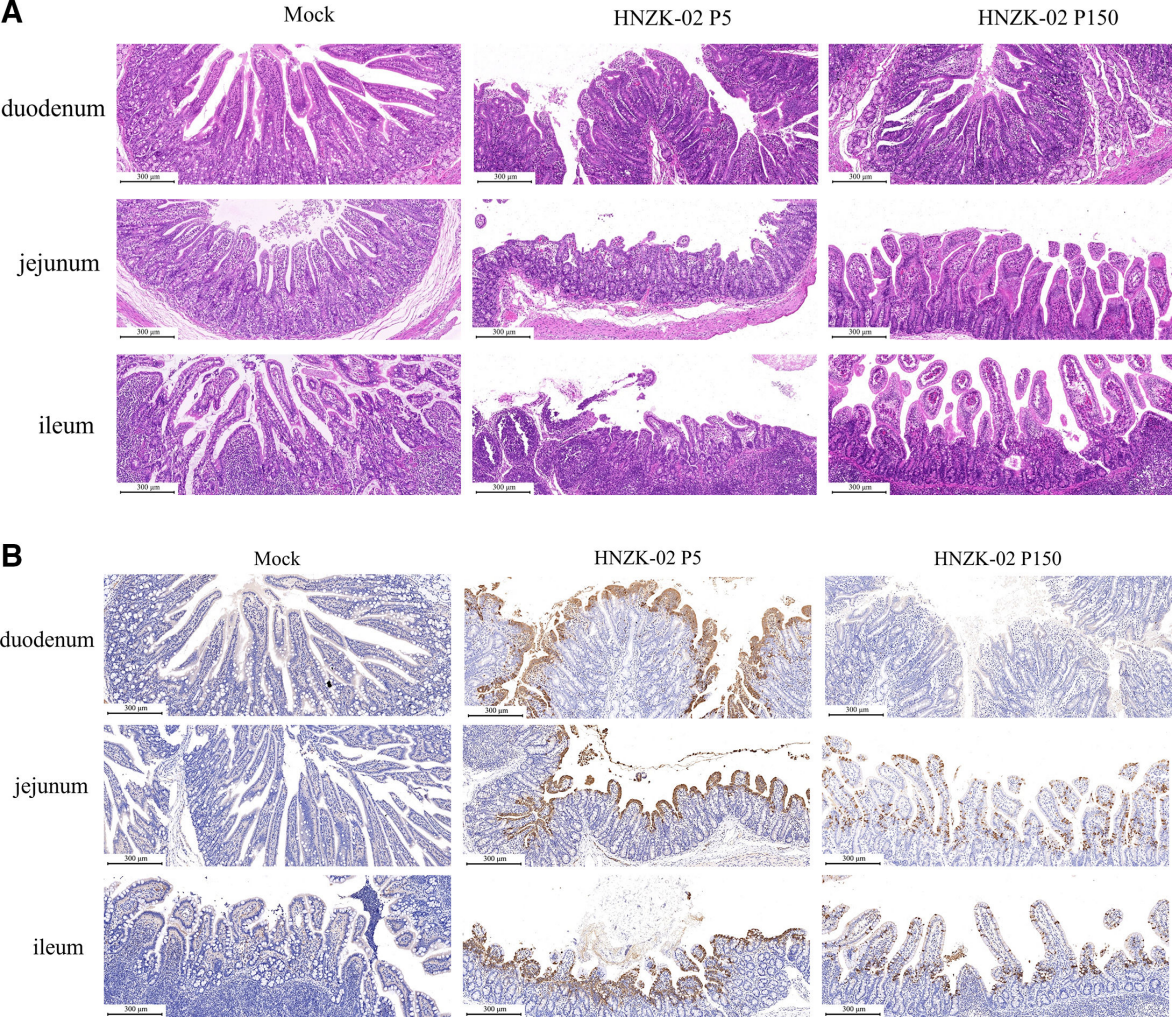
(A and B) Lesions of small intestinal tissue sections from piglets inoculated with PDCoV HNZK-02-P5 or HNZK-02-P150. (**A**) The small intestinal tissues (duodenum, jejunum, and ileum) from the PDCoV HNZK-02-P5- or HNZK-02-P150-infected pigs and control piglets were collected and then stained via the Hematoxylin and eosin (H&E). (**B**) Immunohistochemical analysis of duodenum, jejunum, and ileum were stained with a monoclonal antibody directed against PDCoV N protein. Scale bars are shown in each picture.

The tissues of duodenum, jejunum, and ileum of all piglets were collected for histopathological analysis. As shown in Fig. 6A, in the piglets infected with PDCoV HNZK-02-P5, severe histopathological lesions in all the small intestinal segments were observed, characterized by the villous atrophy and blunting, even shedding of the intestinal villi. In addition, lesions in jejunum were characterized by severe bleeding and inflammatory cell infiltrates. Lesions in ileum were manifested as goblet cell loss. In the piglets infected with PDCoV HNZK-02-P150, no visible microscopic lesions were detected other than slight intestinal villus damage in the duodenum. In the control group, the intestinal villi of the uninfected piglets were intact with no microscopic lesions.

PDCoV antigen was detected in the enterocytes of the duodenum, jejunum, and ileum of the PDCoV HNZK-02-P5- and P150-challenged piglets by the immunohistochemical analysis, whichconsisted with the results of histopathological. The amount of PDCoV detected in jejunum and ileum was significantly higher in the PDCoV HNZK-02-P5-infected group than that in the HNZK-02-P150-infected group. It’s worth noting that no PDCoV-positive enterocytes were detected in the duodenum of the PDCoV HNZK-02-P150-challenged piglets. No PDCoV-positive cells were detected in the control group (Fig. 6B). Taken together, these results further demonstrated that the viral pathogenicity of PDCoV HNZK-02-P150 decreased significantly in 8-day-old piglets.

### Cytokines induced in the jejunum and colon by PDCoV HNZK-02 infection

**Fig 7:**
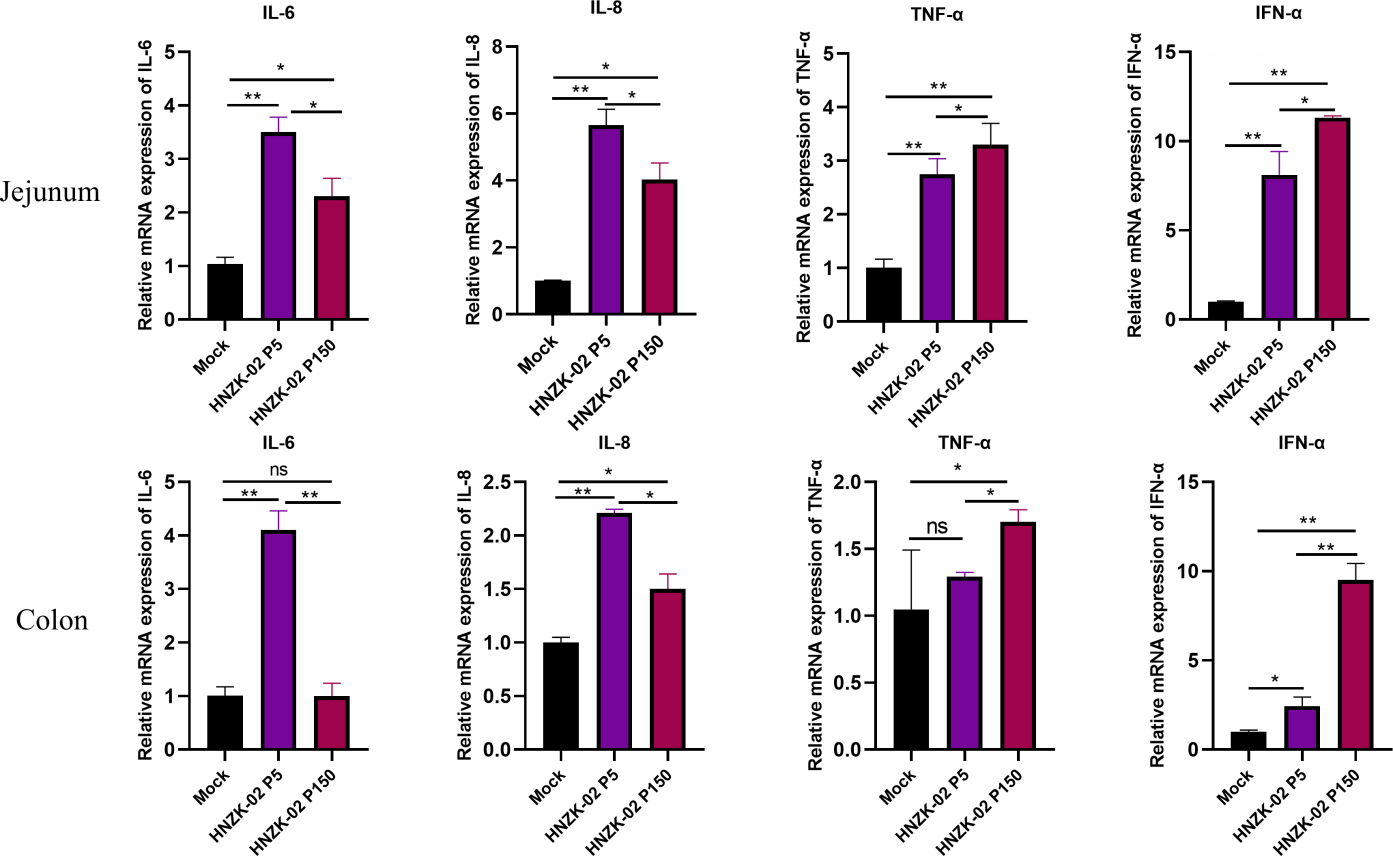
Cytokine detection in the jejunum and colon tissue of piglets inoculated with PDCoV HNZK-02-P5 or HNZK-02-P150. The concentrations of IL-6, IL-8, TNF-α, and IFN-α were measured with qRT-PCR. Error bars indicate the standard deviations from each group (*n* = 3). *P < 0.05; **P < 0.01.

The production of inflammatory cytokines in the target tissue is part of the innate immune response to viral infection (24), the levels of cytokines (interleukin (IL)-6, IL-8, Tumor necrosis factor (TNF)-α, and interferon (IFN)-α) in the jejunum and colon of piglets were detected using the qRT-PCR. In the jejunum and colon, the levels of the IL-6, IL-8, TNF-α, and IFN-α were significantly higher in PDCoV HNZK-02-P5- and HNZK-02-P150-inoculated piglets than in the control group, and the PDCoV HNZK-02-P150 infection induced higher levels of TNF-α (*P* < 0.05) and IFN-α (*P* < 0.01) than those in the PDCoV HNZK-02-P5 infection (*P* < 0.05). The IL-6 and IL-8 secretion in the jejunum and colon of P150-inoculated piglets was decreased significantly (*P* < 0.05), when compared with that of the PDCoV HNZK-02-P5-inoculated or the control piglets (Fig. 7). These results indicated that PDCoV infection could induce the innate immune response in the target tissues of piglets.

### The effects of the virulent and attenuated strains of PDCoV HNZK-02 on the intestinal barrier function of piglets

**Fig 8:**
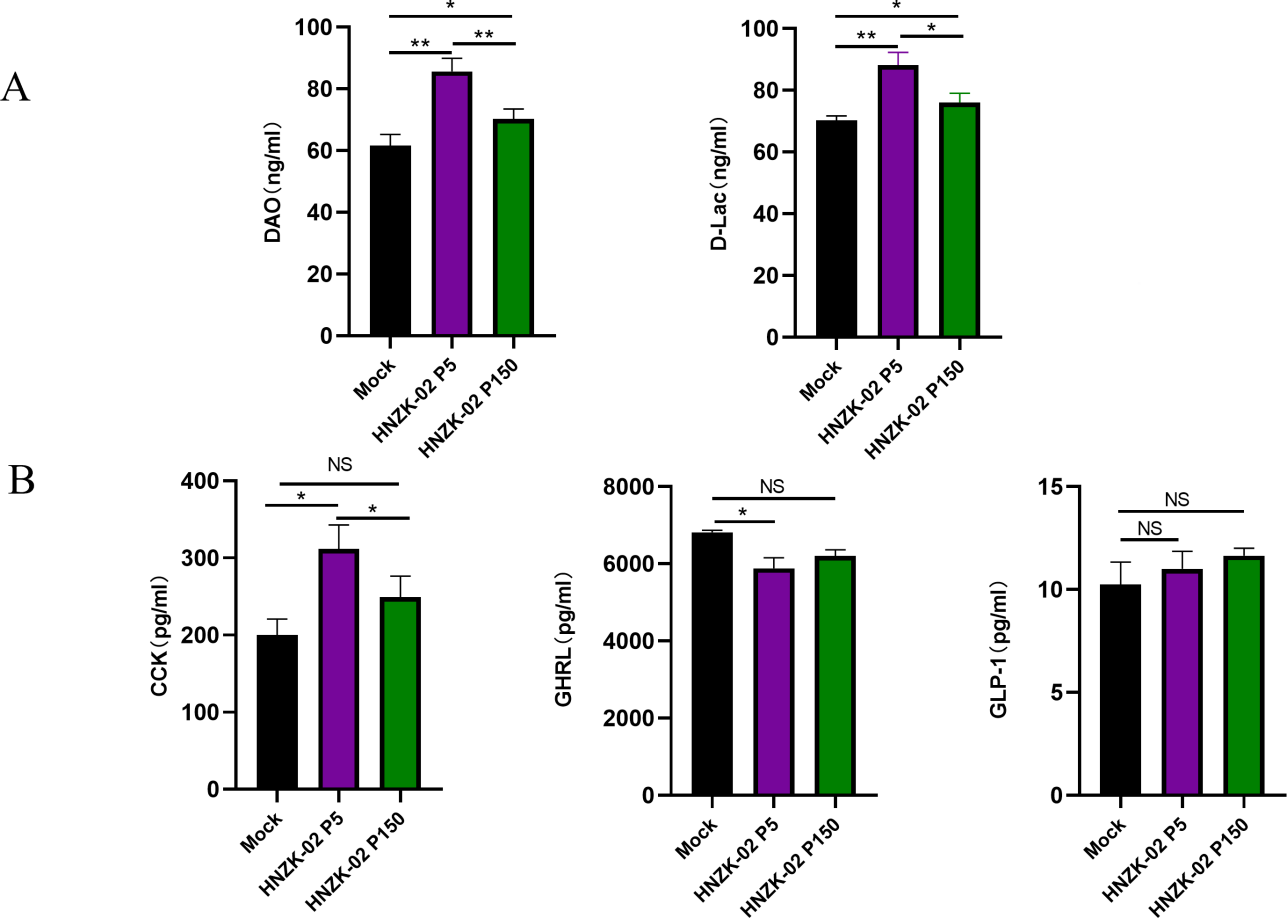
(**A**) The DAO and D-Lac in serums collected from piglets inoculated with PDCoV HNZK-02-P5 or HNZK-02-P150 were detected using enzyme linked immunosorbent assay (ELISA) . (**B**) The cholecystokinin (CCK), ghrelin, and glucagon-like peptide-1 in serums collected from piglets inoculated with PDCoV HNZK-02-P5 or HNZK-02-P150 were detected via ELISA assays at 3 dpi. Error bars indicate the standard deviations from each group (*n* = 3). *P < 0.05; **P < 0.01.

D-lactic acid (D-Lac) and diamine oxidase (DAO) were common indicators of intestinal permeability. The levels of DAO in the serum of the control, PDCoV HNZK-02-P5-inoculated, and PDCoV HNZK-02-P150-inoculated piglets were 61.5 ± 4.0 ng/mL, 85.5 ± 5.0 ng/mL, and 69.2 ± 3.0 ng/mL, respectively. The levels of D-Lac in the ileum of the control, HNZK-02-P5-inoculated, and HNZK-02-P150-inoculated piglets were 68.5 ± 3.0 ng/mL, 87.4 ± 4.0 ng/mL, and 75.6 ± 3.0 ng/mL, respectively. Comparing with the levels of DAO and D-Lac in control piglets, the difference with HNZK-02-P5-inoculated piglets was very significant (*P* < 0.01), and the HNZK-02-P150-inoculated piglets increased significantly (*P* < 0.05) (Fig. 8A). These results demonstrated that the effect of PDCoV HNZK-02 to intestinal permeability of 8-day-old piglets gradually decreased after serial passage *in vitro*.

### The effects of the virulent and attenuated strains of PDCoV HNZK-02 on the growth performance of piglets

Glucagon-like peptide-1 (GLP-1), CCK, and ghrelin (GHRL), which belong to the gut hormones, influence the functioning of the digestive tract and modulate insulin secretion from the pancreas. Simultaneously, these gut hormones also mediate the regulation of food intake by terminating hunger and inducing satiety (43). The levels of CCK in the serum of PDCoV HNZK-02-P5-inoculated piglets (310 ± 30 pg/mL) were increased significantly (*P* < 0.05), and were slightly increased (*P* > 0.05) in the PDCoV HNZK-02-P150-inoculated piglets (240 ± 30 pg/mL), when compared to the control piglets (200 ± 20 pg/mL). The levels of GHRL in the serum of PDCoV HNZK-02-P5-inoculated piglets (5,800 ± 250 pg/mL) were significantly reduced (*P* < 0.05), and were increased (non-significant, *P* > 0.05) in the PDCoV HNZK-02-P150-inoculated piglets (6,250 ± 100 pg/mL), when compared to the control piglets (6,800 ± 70 pg/mL). In addition, there were no obvious changes in the levels of GLP-1 when the PDCoV HNZK-02-P5- and P150-inoculated piglets were compared with the control piglets (Fig. 8B).

### The diversity analysis of colonic microbiota in the PDCoV HNZK-02 virulent and attenuated strains-infected piglets

**Fig 9:**
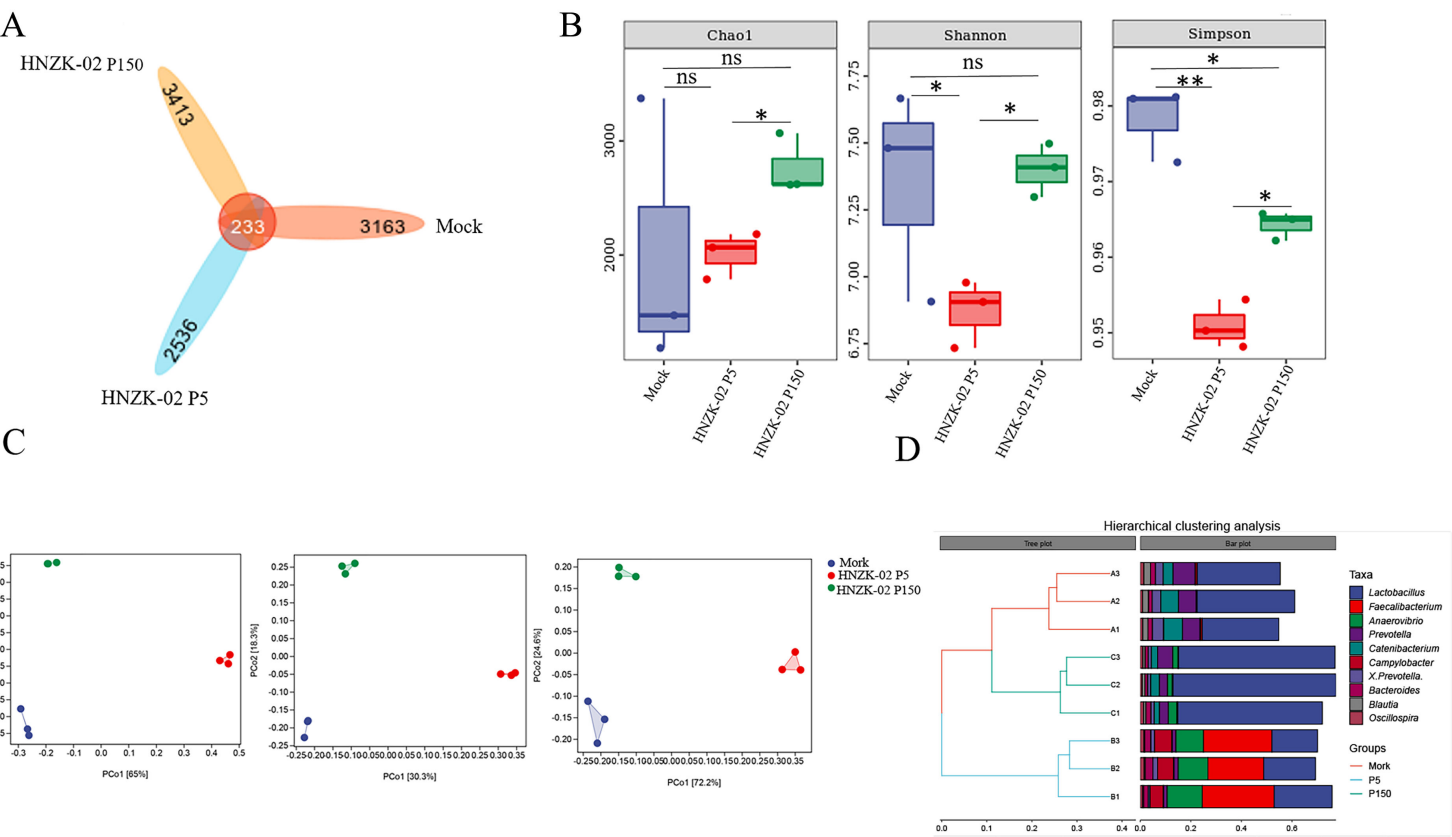
Community structure of colonic microbiota in the PDCoV HNZK-02-P5- or HNZK-02-P150-infected pigs and control piglets. (**A**) Venn diagram of shared OTUs based on the sequences with more than 97% similarity (*n* = 3) in the PDCoV HNZK-02-P5- or HNZK-02-P150-infected pigs and control piglets. (**B**) The alpha diversity indexes (Chao1, Shannon, and Simpson) of the colonic microbiota at the class and order level. (**C**) Principal coordinate analysis of the colonic microbiota based on the Bray-Curtis phylogenetic distance metric (left), unweighted UniFrac (middle), and weighted UniFrac (right). (**D**) The hierarchical clustering analysis of the colonic microbiota. The panel on the left is a hierarchical clustering tree. The composition of the two samples is similar when the branch length between the samples is shorter. The panel on the right is a stacked bar chart of the top 10 genera in abundance. *P < 0.05; **P < 0.01.

The characteristics of the gut microbiota in the control, PDCoV HNZK-02-P5, and HNZK-02-P150-inloculated groups were analyzed by 16S rRNA gene sequencing. Nine hundred fifty-two thousand ninety-seven available sequences were collected and the average length of effective amplicon was 421 bp. Venn diagram showed that the number of operational taxonomic units (OTUs) in control, PDCoV HNZK-02-P5, and HNZK-02-P150 groups was 3,163, 2,536, and 3,413, respectively, and 233 OTUs were shared among the three groups (Fig. 9A). Alpha diversity analysis, including the Chao 1, Shannon, and Simpson diversity indices, showed the mean community richness and microbial diversity were significantly lower in the PDCoV HNZK-02-P5 infection group than that in the control (*P* < 0.05), but there was no significant difference between the control and the PDCoV HNZK-02-P150 group (*P* > 0.05) (Fig. 9B). The results of Beta diversity analysis showed that the microbial composition of colonic in the piglets from the control, PDCoV HNZK-02-P5, and PDCoV HNZK-02-P150 groups could be divided into three different clusters, and the PDCoV HNZK-02-P150 group was closer to the control group (Fig. 9C). Meanwhile, the hierarchical clustering analysis results showed that the control and PDCoV HNZK-02-P150 groups belong to the same subgroup, and the PDCoV HNZK-02-P5 group belong to different branches (Fig. 9D). These results indicated that the structure and composition of the colonic microbial community in the control and PDCoV HNZK-02-P150 groups were relatively close, which was significantly different from the PDCoV HNZK-02-P5 group.

### The changes of gut microbiota structure in the colon of piglets after being infected with the virulent and attenuated strains of PDCoV HNZK-02

**Fig 10:**
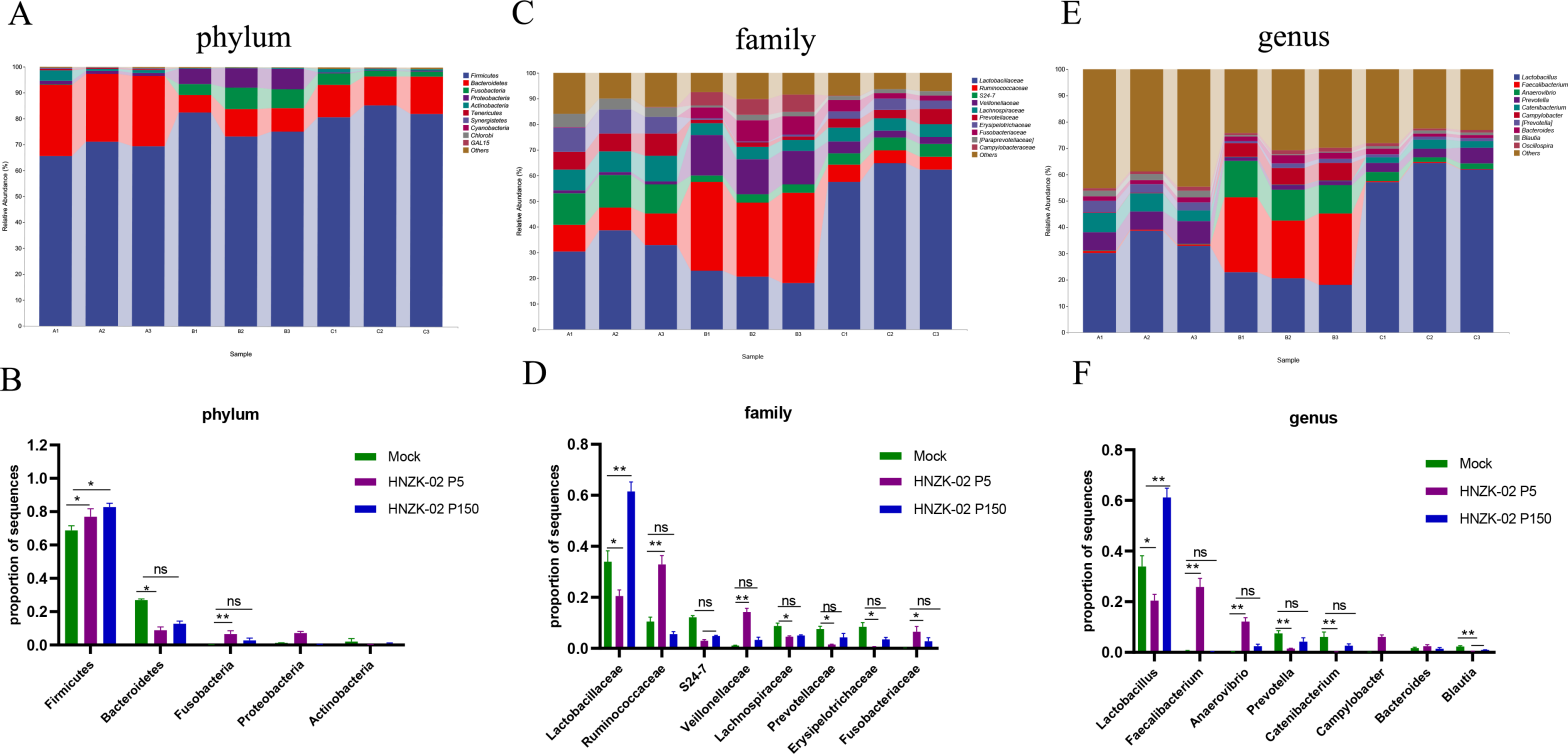
The change of gut microbial composition in piglets after being inoculated with PDCoV HNZK-02-P5 or HNZK-02-P150. The differences in the abundance of bacterial phylum (**A and B**), family (**C and D**), and genus (**E and F**) levels were analyzed using Kruskal-Wallis and Wilcoxon tests. *P < 0.05; **P < 0.01.

To further investigate the microbiota composition and distribution in the colon of the control, PDCoV HNZK-02-P5, and HNZK-02-P150 groups, we analyzed the relative abundance of microbiota at the phylum, family, and genus levels. At the phylum level, *Firmicutes* and *Bacteroidota* were the most predominant phylum in all groups, followed by the *Fusobacteria*, *Proteobacteria*, *Actinobacteria,* and *Tenericutes*. Compared with the control group, the abundance of *Firmicutes* was very significantly increased (*P* < 0.05) in the PDCoV HNZK-02-P150 inoculation group and in the PDCoV HNZK-02-P5 inoculation group. However, the abundance of *Fusobacteria* was very significantly increased in the PDCoV HNZK-02-P5 inoculation group, and there had been no significant difference between the control and PDCoV HNZK-02-P150 inoculation groups (Fig. 10A). At the family level, the PDCoV HNZK-02-P5-infected piglets had higher *Ruminococcaceae*, *Veillonellaceae,* and *Fusobacteriaceae* levels and lower *Lactobacillaceae*, *Lachnospiraceae*, *Prevotellaceae,* and *Erysipelotrichaceae* levels in the colon (*P* < 0.05). Of note, the PDCoV HNZK-02-P150-infected piglets had higher *Lactobacillaceae* levels in the colon (*P* < 0.05) (Fig. 10B). At the genus level, comparing with the control group, *Lactobacillus*, *Prevotella*, *Blautia,* and *Catenibacterium* levels were decreased (*P* < 0.01) and the increasing trend was found for *Faecalibacterium* and *Anaerovibrio* in the colon of the PDCoV HNZK-02-P5-infected piglets. The PDCoV HNZK-02-P150 group had higher levels of members of the *Lactobacillus* and lower levels of members of *Blautia* than the control group (Fig. 10C).

### The analysis of microbiota differences in the colon across all the pig groups

**Fig 11:**
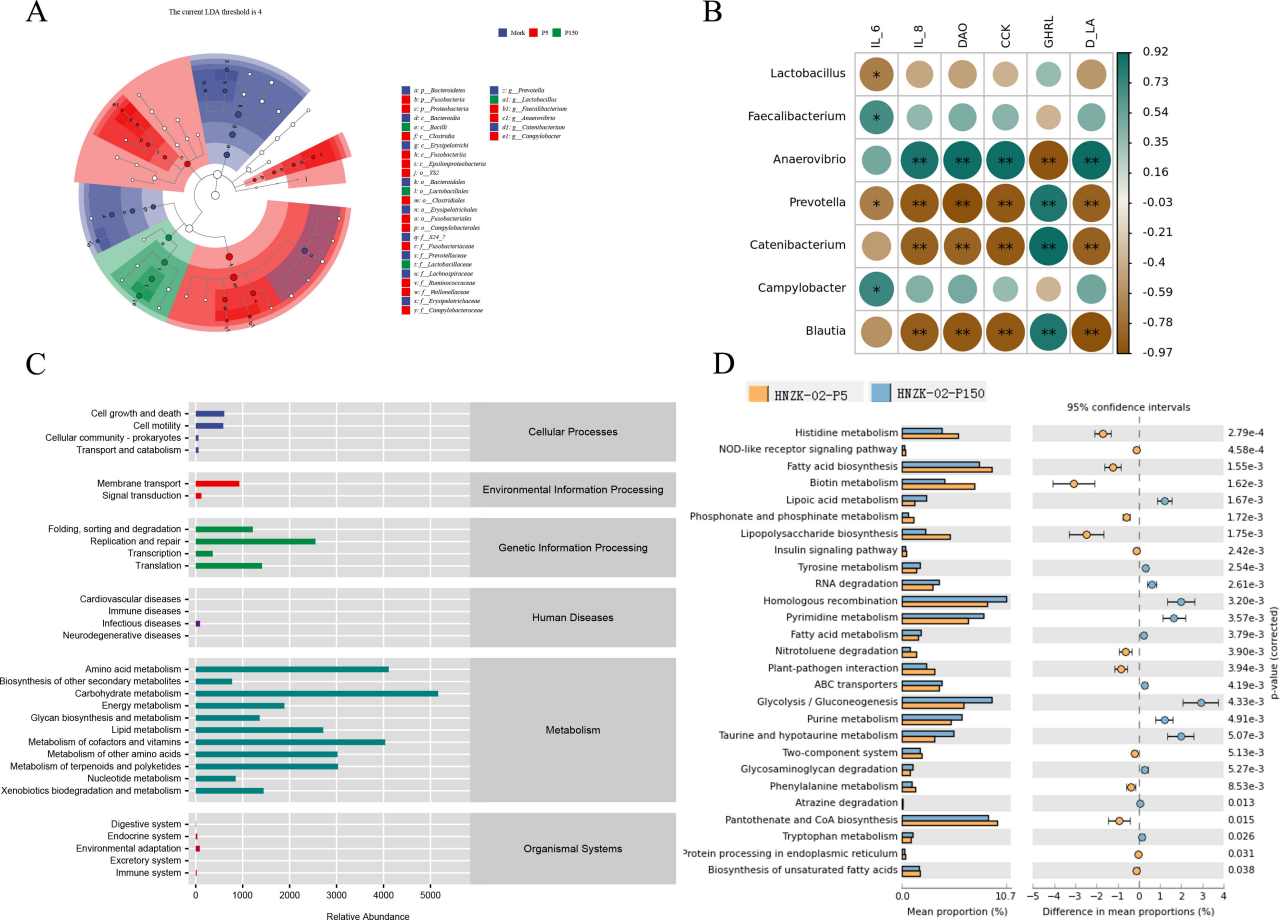
(**A**) The cladogram of enriched taxa based on LEfSe analysis reveals significant differences of the colonic microbial community between groups. Only taxa meeting a linear discriminant analysis significant threshold >3 are shown (p, phylum level; c, class level; o, order level; f, family level; g, genus level). (**B**) Heatmap of the correlation analysis between microbiota composition and inflammatory factors in the colon or hormone in serum. Green indicates a positive correlation and brown indicates a negative correlation. (**C**) Functional metagenomics prediction analysis of gut microbiota using the PICRUSt. (**D**) Differences in the predictive functions are tested using White’s non-parametric *t*-test of the Statistical Analysis of Metagenomics Profiles software. The deeper the color means the greater the correlation. *P < 0.05; **P < 0.01.

Linear discriminant analysis effect size (LEfSe) analysis was used to determine and distinguish the composition of the gut microbiota between the control, PDCoV HNZK-02-P5-, and PDCoV HNZK-02-P150-infected piglets. There were significant differences on microbiota compositions in the colon between the control, PDCoV HNZK-02-P5-, and PDCoV HNZK-02-P150-infected piglets (Fig. 11A). As shown in Table 1, 11 potential microbial biomarkers were identified in the control group, which dominated by *Prevotellaceae*, *Lachnospiraceae,* and *Erysipelotrichaceae*. The gut microbiota of the PDCoV HNZK-02-P150 group included four microbial biomarkers and was predominated by *Lachnospiraceae*, whereas the microbiota of PDCoV HNZK-02-P5 group had 15 identified microbial biomarkers and was dominated by the *Ruminococcaceae*, *Veillonellaceae,* and *Campylobacteraceae*.

**TABLE 1:** The microbial biomarkers identified in the control, HNZK-02-P5-, and HNZK-02-P150-infected piglets by the LEfSe analysis

Control group	HNZK-02-P5 infection group	HNZK-02-P150 infection group
*Bacteroidetes*	*Fusobacteria*	*Bacilli*
*Bacteroldia*	*Proteobacteria*	*Lactobacillales*
*Erysipelotrichi*	*Clostridia*	*Lactobacillaceae*
*Bacteroidales*	*Fusobacterlia*	*Lactobacillus*
*Erysipelotrichales*	*Epsilonproteobacteria*	
*S24_-_7*	*Clostridiales*	
*Prevotellaceae*	*Fusobacteriales*	
*Lachnospiraceae*	*Campylobacterales*	
*Erysipelotrichaceae*	*Fusobacteriaceae*	
*Prevotella*	*Ruminococcaceae*	
*Catenibacterlum*	*Veillonellaceae*	
	*Campylobacteraceae*	
	*Faecalibacterium*	
	*Anaerovibrio*	
	*Campylobacter*	

### Relationship between microbial signatures and clinical indexes in piglets

Spearman analysis was conducted to evaluate the correlation between predominant bacteria and clinical indexes, including GLP-1, GHRL, CCK, DAO, D-Lac, IL-6, IL-8, TNF-α, and IFN-α in PDCoV HNZK-02-P5- and P150-infected piglets, respectively. As shown in Fig. 11B, Compared with control, the *Lactobacillus* and *Prevotella* showed a negatively correlation with the IL-6 (*P* < 0.05) expression levels and the *Faecalibacterium* and *Campylobacter* were positively correlated with IL-6 expression level. The *Catenibacterium*, *Prevotella,* and *Blautia* were positively correlated with the GHRL expression level (*P* < 0.01), and negatively correlated with IL-8, DAO, D-Lac, and CCK expression levels (*P* < 0.01). In addition, there was a positive correlation between *Anaerovibrio* and the IL-8, DAO, D-Lac, and CCK expression levels (*P* < 0.01), and a negative correlation with the GHRL expression level (*P* < 0.01) (Fig. 11B).

### Functional metagenomics prediction

To demonstrate that PDCoV HNZK-02-P150-induced microbial changes could modulate the metabolic function of gut microbiota, we conducted functional metagenomics prediction of gut microbiota based on 16S rRNA gene sequencing using the (phylogenetic investigation of communities by reconstruction of unobserved states) PICRUSt and these pathways detected in HNZK-02-P5 and HNZK-02-P150 infection groups involved in cellular processes, environmental information processing, genetic information processing, human diseases, metabolism, etc (Fig. 11C). Then, we tested the differences of these pathways using the Statistical Analysis of Metagenomics Profile software; our analysis demonstrated significantly higher proportions of “tryptophan metabolism,” “lipoic acid metabolism,” “pyrimidine metabolism,” and “tyrosine metabolism” together with a significantly lower proportion of “NOD-like receptor signaling pathway,” “riboflavin metabolism,” “fatty acid biosynthesisin,” and “lipopolysaccharide biosynthesis” in the PDCoV HNZK-02-P150 group (Fig. 11D).

## DISCUSSION

The newly emerged coronavirus PDCoV is an enteropathogen causing severe diarrhea, dehydration, and death in nursing piglets, devastating great economic losses for the global swine industry (44, 45). Furthermore, it can infect various avian and mammalian species (18, 20, 46). The report showed that PDCoV can infect humans and cause an acute undifferentiated febrile illness in children in Haiti (21). These evidences indicated that PDCoV has cross-species transmission and zoonotic potential (16). There are currently no approved treatments or vaccines available for PDCoV. Consequently, the development of the PDCoV live-attenuated vaccines is crucial for the prevention and control of PDCoV infection. In our current study, our previously isolated PDCoV HNZK-02 strain (41) was passaged over 150 times using LLC-PK cells to develop an attenuated PDCoV strain. The sensitivity and adaptability of PDCoV HNZK-02 in LLC-PK cells increased gradually with the serial passaged. The PDCoV strain HNZK-02-P150 showed no obvious clinical signs, low fecal virus shedding, and mild histopathology in the intestine of 8-day-old piglets, indicating the PDCoV HNZK-02 has been attenuated by cell culture passage, and it may be a potential vaccine candidate to establish a novel attenuated vaccine; however, the immunogenicity in pregnant sows and the protective efficacy in the piglets of this strain should be assessed before its preparation as an attenuated vaccine.

Previous study showed that the virulence of the virus can be reduced via serial passage *in vitro*; the virulence of the high-passage PEDV FJzz1 variants, JS-2/2014 and PC22A were markedly reduced in piglets (24, 47, 48). In the present study, the disease was reproduced experimentally in 8-day-old piglets using PDCoV HNZK-02-P5, with the symptoms of acute watery diarrhea and severe intestinal lesions. However, PDCoV HNZK-02-P150-infected piglets had no obvious clinical signs and slightly intestinal lesions throughout the whole experiment, indicating the high-passage PDCoV HNZK-02-P150 was higher degree of attenuation *in vivo*. The S protein of CoVs is the pivotal surface glycoprotein that mediated viral attachment and entry host cells (49). Simultaneously, the S protein is the key surface glycoprotein involved in virus attenuation and induction of neutralizing antibodies *in vivo* (48, 50). Hence, it is a critical determinant of viral host range and tissue tropism, and also most of the host immune responses induction (51). The Aa changes of PDCoV HNZK-02 variants P60, P100, and P150, relative to the P5 strain, mainly occurred in the S glycoproteins, and the partial mutations in the S1 of PDCoV HNZK-02-P150 caused its structure to change. We speculated that these Aa changes could cause the different pathogenicity of PDCoV HNZK-02-P5 and HNZK-02-P150, which required our further experiments to confirm using reverse genetics analysis.

As a candidate vaccine, the effects of growth performance on the piglets should also be evaluated. GLP-1 and CCK are secreted by enteroendocrine cells, and their plasma concentrations increase in response to feed intake (52). In addition, mammalian GHRL is a potent stimulator of growth hormone release and enhances feeding and weight gain to regulate energy balance (53). In this study, the infection of PDCoV HNZK-02-P5 promoted CCK level and decreased GLP-1 levels in serum, and inhibited short-term feed intake in piglets, which resulted that the body weight in PDCoV HNZK-02-P5 inoculation group was decreased, but the HNZK-02-P150-inoculated piglets remained relatively stable. These results demonstrated that PDCoV HNZK-02-P150 strain is safe for piglets, and it may be exploited as a vaccine candidate for PDCoV. Moreover, DAO is an intracellular enzyme abundant in the epithelium of the small intestine, while D-Lac is a product of intestinal bacteria released into the blood during villi injury. Moreover, we found that PDCoV HNZK-02-P5 infection resulted in an increase in intestinal mucosal permeability characterized by the high levels of serum DAO and D-Lac, suggesting that the piglets’ intestinal barrier function was compromised, while the levels of DAO and D-Lac in the PDCoV HNZK-02-P150-inoculated piglets were low, indicating that the damage of intestinal barrier for PDCoV was reduced gradually as this virus was serially passaged.

The pro-inflammatory cytokines such as IL-6, IL-8, TNF-a, and IFN-a play pivotal roles in the antiviral response (24, 54). Several previous studies have confirmed that PEDV and PDCoV E proteins can significantly activate nuclear factor kappa-B (NF-κB) which consequently promotes IL-8 expression (55, 56). While the PEDV ORF3 can inhibit cellular IL-6 and IL-8 production by blocking the NF-κB p65 activation (57). In our previous experiments, PDCoV infection has caused the excessive secretion of pro-inflammatory cytokines (IL-6 and IL-8) and further mediated piglet intestinal pathological lesions. In this study, the IL-6 and IL-8 productions in the jejunum and colon were also significantly up-regulated in PDCoV-infected piglets, indicating that these cytokines were involved in the induction of intestinal lesions during PDCoV infection. The lower concentration of IL-6 and IL-8 induced by the attenuated PDCoV HNZK-02-P150 infection may also have contributed to the less lesions in piglets. Previous research showed that the high level of TNF-α can protect against influenza infection. In the present study, PDCoV HNZK-02-P150 infection could cause the higher levels of TNF-α in jejunal and colon tissues relative to those in the PDCoV HNZK-02-P5 group. Moreover, the type I IFN-mediated antiviral response is an important component of virus-host interactions and plays an essential role in inhibiting virus infection (58). In this study, the PDCoV HNZK-02-P150 infection induced high levels of IFN-α transcription than PDCoV HNZK-02-P5. Previous studies have reported that PDCoV infection inhibits the type I IFN response to evade the host’s antiviral immune responses (59–61). Therefore, we hypothesized that the high level of TNF-α and IFN-α caused by the attenuated PDCoV HNZK-02-P150 infection might activate the host immune defenses and inhibit PDCoV proliferation in the target tissues, which is consistent with the attenuated pathogenicity of PDCoV HNZK-02-P150 in piglets.

The relationship between gut microbiota and viral diseases has been a research hotpot in recent years. When the host was infected with pathogens, the species and abundance of the gut microbiota would be changed significantly, characterized by increasing pathogenic bacteria and decreasing normally dominant bacteria (62). In our study, PDCoV HNZK-02-P5-inoculated piglets had lower levels of alpha diversity and beta diversity of gut microbiota than the control and P150-inoculated piglets. In addition, at the phylum level, *Firmicutes* was significantly increased in piglets of P150 inoculation compared to the P5-inoculated and control piglets. *Firmicutes* can produce large amounts of lactic acid and butyrate to promote the development of intestinal epithelial cells and protect the intestinal tract from infection (63). These results suggested that the host is changing the gut microbiota to resist PDCoV infection; the higher abundance of *Firmicutes* in P150-inoculated piglets might activate the immune defenses to protect against PDCoV infection. At the family level, the *Lachnospiraceae* can play important roles in maintaining gut health by fermenting carbohydrates and producing butyrate (64)*, Veillonellaceae* and *Fusobacteriaceae* are strict anaerobes and detrimental bacterial families (65). *Lachnospiraceae* significantly decreased and *Veillonellaceae* and *Fusobacteriaceae* significantly increased after PDCoV HNZK-02-P5 infection, suggesting that the infection with PDCoV HNZK-02-P5 could decrease normally dominant bacteria and increase pathogenic bacteria. At the genus level, *Anaerovibrio*, *Catenibacterium,* and *Blautia* were significantly increased in PDCoV HNZK-02-P5-infected piglets. At the same time, previous study reported that the gut microbiota changed with virus infection may involve in the intestinal immune response (66). We previously found the *Eisenbergiella* is strongly correlated with the levels of TNF-α and IFN-γ in chickens (67), and *Peptostreptococcus* and *Mitsuokella* had a strong positive correlation with TNF-α, IL-6, and IL-8 secretion in piglets infected with PDCoV (68). In this study, *Lactobacillus* showed a negative correlation with the IL-6 expression levels and *Faecalibacterium* and *Campylobacter* were positively correlated with IL-6 expression level. The IL-8 secretion is strongly correlated with *Catenibacterium*, *Prevotella*, *Blautia,* and *Anaerovibrio*. The change of microbiota was closely related to the level of cytokines (directly or indirectly, positively or negatively).

Notably, *Lactobacillus*, which belong to the gut microbiota was useful to maintain the gut microbiota balance and intestinal function (69, 70). In this study, *Lactobacillus* was significantly decreased after PDCoV HNZK-02-P5 infection, and significantly increased after PDCoV HNZK-02-P150 infection. Previous research indicated that the variety of *Lactobacilli* can modulate the gut microbial composition and result in improved gut health (71), and oral administration of *Lactobacillus* can reduce potential enter pathogens in weaning and growing-finishing pigs (72, 73). Therefore, we conjectured that the increased abundance of *Lactobacillus* could regulate and improve the gut microbial destroyed by the PDCoV, stimulate the immune system to resist the infection of PDCoV, which is consistent with the attenuated pathogenicity of PDCoV HNZK-02-P150 in piglets. Simultaneously, a higher diversity in the gut microbiota could improve protection from *Shigella dysenteriae* infection of attenuated *S. dysenteriae* vaccines (74). It has been reported that gut bacteria can affect the effect of the vaccine; a higher relative abundance of the phylum *Firmicutes* and *Bacteroidetes* was associated with both humoral and cellular vaccine responses (75, 76). In our study, *Firmicutes* was significantly increased and *Bacteroidetes* was significantly reduced in HNZK-02-P150-infected piglets when compared with the control piglets. These results suggested that as a candidate vaccine, the changes of gut microbiota induced by PDCoV HNZK-02-P150 may stimulate its induced immune effects, and improved protection from PDCoV infection. Nevertheless, we need further research studies to completely understand the interaction molecular mechanism of the virus infection, vaccine immunity, and gut microbiota.

To further investigate the change in metabolic function of the gut microbiota, functional metagenomics prediction analysis was performed using PICRUSt. Our analysis demonstrated significantly higher proportions of “tryptophan metabolism” and “lipoic acid metabolism,” together with a significantly lower proportion of “NOD-like receptor signaling pathway” and “lipopolysaccharide biosynthesis” in the PDCoV HNZK-02-P150 group. Studies have shown that metabolites derived from tryptophan could regulate the inflammation and disease development such as limited activation of NF-κB, a transcription factor that drives the production of pro-inflammatory cytokines (77), and the lipopolysaccharide is harmful for the maintenance of physiological homeostasis (78). Moreover, The NOD-like receptors are a family of pattern recognition receptors expressed in a variety of tissue types, and have been reported to regulate cell pathways that govern both the growth and the immune response to stimuli, including the mitogen-activated protein kinases (MAPK) and NF-κB pathways (79). These alterations in predictive functions were also consistent with the low pathogenicity of PDCoV HNZK-02-P150, including the lower inflammatory response and intestinal permeability.

PEDV, TGEV, and PDCoV could cause acute gastroenteritis in piglets, characterized by diarrhea, vomiting, and dehydration, which also cause huge losses to the pig industry worldwide (4). The vaccines are the most effective interventions to prevent and control viral transmission (22). Moreover, gut microbiota has an important relationship with the development of many diseases (29). The study validated the point that the virulence of the virus can be reduced via serial passage *in vitro*, proposing the point that gut microbiota can regulate the pathogenicity of PDCoV to piglets, which provide a basis for the development of new methods and strategies for effective prevention and control of other porcine enteric coronaviruses, including PEDV, TGEV, and PDCoV. The development of live-attenuated vaccine and next-generation probiotics are conducive to the prevention and control of enteric-associated viruses and reduce the economic loss of aquaculture industry.

In summary, our research successfully generated an attenuated PDCoV variant strain HNZK-02 by serial passage on LLC-PK cells. And the pathogenicity of this attenuated strain was evaluated in 8-day-old piglets. The viral load in the intestines and fecal viral shedding of PDCoV HNZK-02-P150-infected piglets were significantly lower than that in the PDCoV HNZK-02-P5-infected piglets. The changes in genomic composition and structures of PDCoV, and the production of the pro-inflammatory cytokines and the changes in gut microbiota and metabolic function in piglets, might account for the underlying molecular mechanisms of PDCoV attenuation. We speculated that the PDCoV HNZK-02-P150 may be an attenuated vaccines for developing PDCoV, and we need further research to explore the interaction molecular mechanism of viral infection, vaccine immunity, and gut microbiota regulation of this attenuated PDCoV strain.

## MATERIALS AND METHODS

### Virus serial propagation in LLC-PK cells

The LLC-PK cells were used to propagate the PDCoV HNZK-02 in T_25_ flasks. Briefly, the cells were seeded into the T_25_ cell culture flasks and grown to 80%−90% confluency after 24 h. Cells were washed with D-Hanks, and then incubated with the PDCoV HNZK-02 isolated and identified by our laboratory (41). After adsorption for 2 h, the cells were washed with D-Hanks, and the 4–5 mL of maintenance medium supplemented with 5 µg/mL of trypsin (Sigma-Aldrich) was added. The flasks were incubated at 37°C in 5% CO_2_, and when over 80% CPE was evident in the vial-inoculated cell monolayers, the flasks were frozen at −80°C and thawed twice. The cells and supernatants were harvested together and the virus titration was performed by TCID_50_ assay (80).

### Viral replication kinetics in LLC-PK cells

When the LLC-PK cells in six-well plates reached 90%–100% confluence, the cells were inoculated with PDCoV HNZK-02-P5, P30, P60, P100, and P150 strains at MOI of 0.01, respectively. Cells and supernatants were harvested together at 6, 12, 24, 36, 48, 60, and 72 hpi. Subsequently, virus RNA and infectious virus titer were determined by qRT-PCR and TCID_50_ assay, respectively (80).

### Complete genomic analysis of the cell culture-adapted PDCoV HNZK-02 strains

The complete genomes of PDCoV HNZK-02-P60, P100, P120, and P150 were sequenced using 13 primer pairs based upon our previous report (81), and deposited in the GenBank database with the numbers of ON402372, ON382562, OR122653, and OR122654. The genomic fragments were sequenced, and then assembled and analyzed using DNA Star Lasergene 7.0. Then the phylogenetic tree was constructed using the maximum likelihood method with MEGA 6.06. (https://www.megasoftware.net/) based on the S genes from PDCoV HNZK-02-P60, P100, P120, and P150 together with other 54 reference PDCoV strains in GenBank. Three-dimensional structures for the S proteins of PDCoV HNZK-02-P5 and P150 were predicted using Phyre2, and the predicting results were visualized using the PyMol software.

### Pathogenicities evaluation of PDCoV HNZK-02-P5 and P150 strains

Thirteen 8-day-old healthy Duroc × Landrace × Yorkshire piglets with similar body weight were purchased from a commercial pig farm in Henan Province, China. Before viral inoculation, the blood and rectal swabs were collected from all the piglets for the detection of the common diarrhea-related viruses, including PDCoV, TGEV, PEDV, porcine sapelovirus, porcine circovirus type 2, and porcine reproductive and respiratory syndrome virus, with viral-specific PCRs (18). The piglets were divided randomly into three groups (*n* = 4), including the PDCoV HNZK-02-P5-infected group, the HNZK-02-P150-infected group, and the control group. The piglets in different groups were individually housed and the diet was designed to meet requirements recommended by the National Research Council throughout the process.

### Histopathology and immunohistochemistry

Duodenum, jejunum, and ileum were fixed in 4% paraformaldehyde for 24–48 h at room temperature and the histopathology and immunohistochemistry were performed according to our previous reported method (82). The detection of PDCoV antigens was performed using anti-PDCoV-N protein-specific monoclonal antibody (prepared in our lab), followed by incubation with horseradish peroxidase-conjugated goat anti-mouse IgG secondary antibody (Sigma-Aldrich).

### Cytokines detection in swine jejunum and colon tissue using qRT-PCR

The levels of IL-6, IL-8, IFN-α, and TNF-α in swine jejunum and colon tissue were detected using qRT-PCR. qRT-PCR was performed using SYBR Green PCR Master [Takara Biotechnology (Dalian) Co., Ltd., Japan] and the primers used were listed in Table 2. β-Actin was used as the internal control and the date are expressed as fold differences between control and infected pigs using the 2^-ΔΔCT^ method.

**TABLE 2:** The sequences of primers used in this study for real-time RT-PCR

Primers	Sequences (5´−3´)	Product (bp)
IL-6-F	TTCCAGCCCTCCTTCCTG	94
IL-6-R	AGGTCCTTGCGGATGTCG	
IL-8-F	TTCAGTCCAGTCGCCTTCT	98
IL-8-R	GTGGCATCACCTTTGGCATCTTCTT	
TNF-α-F	CCCTTGAGCATCAACCCT	131
TNF-α-R	GCATTGGCATACCCACTCT	
IFN-α-F	CATCCTGGCTGTGAGGAAATAC	127
IFN-α-R	CAGGTTTGTGGAGGAAGAGAAG	
β-Actin-F	TTCCAGCCCTCCTTCCTG	94
β-Actin-R	AGGTCCTTGCGGATGTCG	

### Gastrointestinal function and intestinal permeability detection using ELISA

To determinate the effects of PDCoV infection on gastrointestinal function and intestinal permeability of piglets, the expression levels of the GHRL, GLP-1, CCK, DAO, and D-Lac in serum were detected using ELISA Kits (mIBio, China). Sera from all the experimental piglets were separated from blood and then stored at −20°C before testing. According to the manufacturer’s instructions, the concentrations of GHRL, GLP-1, CCK, DAO, and D-Lac in serum were quantitated using a standard curve.

### DNA extraction and 16S rRNA gene amplicon sequencing

Colonic content samples were collected from all experimental piglets. The total genomic DNA was extracted with OMEGA Soil DNA Kit (M5635-02) (Omega, USA), and stored at −20°C before testing. The NanoDrop NC2000 spectrophotometer (Thermo Fisher Scientific, USA) was used to measure the quantity of extracted DNAs. DNA was amplified by the PCR of the V3-V4 region of bacterial 16S rRNA genes. The Quant-iT PicoGreen dsDNA Assay Kit (Invitrogen, USA) was used to quantified the PCR amplicons. After the individual quantification step, amplicons were pooled in equal amounts, and the pair-end 250 bp sequencing was performed with the Illumina NovaSeq platform with NovaSeq 6000 SP Reagent Kit (500 cycles) at Shanghai Personal Biotechnology Co., Ltd (China).

### Sequence analysis

Raw fastq files were demultiplexed, quality-filtered by Trimmomatic, and merged by FLASH (83). For further analysis, we obtained the effective tags after data filtration and chimera removal. Microbiome bioinformatics were performed with QIIME2 2019.4 with slight modification according to the official tutorials (https://docs.qiime2.org/2019.4/tutorials/) (84). Briefly, the high-quality sequences were assigned to samples according to barcodes. QIIME2 is used to cluster high-quality readings into the OTUs, and the GreenGene Database was used to annotate taxonomic information for each representative sequence. The OTU with 97% similarity is used for Venn diagram and alpha diversity (Chao, Simple, and Shannon) analysis. Beta diversity was determined using principal coordinate analysis based on Bray-Curtis distance analysis, which was conducted to assess the relationships among the different groups. Bacterial taxa leading to differences between groups were identified by LEfSe (85), and the threshold of linear discriminant analysis is 4. For details of 16S rRNA sequencing data analysis, PICRUSt was used to predict the functional profiles of gut microbiota.

### Correlation analysis

Spearman analysis was conducted to evaluate the correlation between predominant bacteria and clinical indexes, including GLP-1, GHRL, CCK, DAO, D-Lac, IL-6, IL-8, TNF-α, and IFN-α in PDCoV HNZK-02-P5- and PDCoV HNZK-02-P150-infected piglets, respectively. The significance thresholds |*R*| > 0.6 and *P*-values lower than 0.05 were considered as having a correlation.

### Statistical analysis

Statistical analyses were performed with SPSS 24.0 software and charts were generated using the GraphPad Prism 8.0 software. The comparison between two groups was identified using a Student’s *t*-test, the multiple comparisons were used by one-way analysis of variance software, and the significant differences in the data microbiome compositions between groups were tested by Kruskal-Wallis and Wilcoxon tests. Significant differences are considered significant at *P < 0.05 and **P < 0.01.

## Data Availability

All data and the code used to reanalyze the data reported in this paper is available from the corresponding author upon request, and data on gut microbiota are publicly available at PRJNA997708.
